# Profiling and Functional Analysis of long non-coding RNAs in yak healthy and atretic follicles

**DOI:** 10.1590/1984-3143-AR2021-0131

**Published:** 2022-10-24

**Authors:** Yilong Yao, Zhaoyi Meng, Wangchang Li, Yefen Xu, Yunlu Wang, Sizhu Suolang, Guangyin Xi, Lei Cao, Min Guo

**Affiliations:** 1 Animal Science Department, Tibet Agriculture & Animal Husbandry College, Nyingchi, Tibet, China; 2 Genome Analysis Laboratory of the Ministry of Agriculture, Agricultural Genomics Institute at Shenzhen, Chinese Academy of Agricultural Sciences, Beijing, China; 3 Provincial Key Laboratory of Tibet Plateau Animal Epidemic Disease Research, Tibet Agriculture & Animal Husbandry College, Nyingchi, Tibet, China; 4 College of Animal Sciences and Technology, China Agricultural University, Haidian, Beijing, China

**Keywords:** yak, RNA-seq, healthy follicles, atretic follicles, lncRNA

## Abstract

Yak is the livestock on which people live in plateau areas, but its fecundity is low. Follicular development plays a decisive role in yak reproductive performance. As an important regulatory factor, the expression of long non-coding RNA (lncRNAs) in yak follicular development and its regulatory mechanism remains unclear. To explore the differentially expressed lncRNAs between healthy and atretic follicular in yaks. We used RNA-seq to construct lncRNA, miRNA, and mRNA expression profiles in yak atretic and healthy follicles, and the RNA sequence results were identified by qPCR. In addition, the correlation of lncRNA and targeted mRNA was also analyzed by Starbase software. Moreover, lncRNA/miRNA/mRNA networks were constructed by Cytoscape software, and the network was verified by dual-luciferase analysis. A total of 682 novel lncRNAs, 259 bta-miRNAs, and 1704 mRNAs were identified as differentially expressed between healthy and atretic follicles. Among them, 135 mRNAs were positively correlated with lncRNA expression and 97 were negatively correlated, which may be involved in the yak follicular development. In addition, pathway enrichment analysis of differentially expressed lncRNA host genes by Kyoto Genome Encyclopedia (KEGG) showed that host genes were mainly involved in hormone secretion, granulosa cell apoptosis, and follicular development. In conclusion, we identified a series of novel lncRNAs, constructed the lncRNA ceRNA regulatory network, and provided comprehensive resources for exploring the role of lncRNAs in yak ovarian follicular development.

## Introduction

As a special economic animal, the yak provides a variety of materials for people in the plateau area. However, the singleton is an important factor restricting the development of the yak industry. The healthy development of follicles is the decisive factor in yak reproduction. Studies have shown that female mammals begin to form the earliest primitive follicles during embryonic development (Yoshimura and Barua, 2017). There are lots of follicles in the ovaries before and after birth. But, most follicles become atretic and degenerate after sexual maturity, and only a few follicles mature and ovulate (Yoshimura and Barua, 2017; Murdoch and McCormick, 1992). Therefore, it is urgent to identify new regulatory factors and mechanisms that regulate follicular maturation and atretic in yaks.

A large number of studies have shown that miRNA, as a post-transcriptional regulatory factor, has been reported to be involved in regulating the development of follicular. For example, miR-21 (Li et al., 2019), miR-181a (Zhang et al., 2017), miR-644-5p (Sun et al., 2019) and let-7g (Zhang et al., 2019a) affect follicular development by regulating GCs apoptosis proliferation and autophagy. LncRNA is another important noncoding regulatory RNA that was reported to play an indispensable role in the formation of early germ cells, the implantation and development of early embryos, and the regulation of hormones (Vasconcelos et al., 2018; Yan et al., 2013). By comparing human, mouse, and other fertilized eggs with gametes before fertilization and early embryos at different development stages, scientists found a large number of lncRNAs associated with different embryonic development stages (Hamazaki et al., 2015; Caballero et al., 2014), such as *lncRNA-MEG3* interacting with *JARID2* to recruit *PRC2* inhibition of gene expression related to embryonic development by trans-action (Kaneko et al., 2013). Chen et al. found that 24 lncRNAs were differentially expressed in ovaries at different stages of mouse embryonic development, and 147 lncRNAs were differentially expressed in male and female reproductive organs with the same gestational age (Chen et al., 2012). Brown et al. found a large number of promoter-related antisense lncRNAs in drosophila and mouse ovaries, and these lncRNAs may regulate the transcriptional activation of their homologous genes (Brown et al., 2014). However, it is rare to explore the function of lncRNA in livestock reproduction by transcriptome sequencing. Most of these studies focus on pigs, sheep, and chickens. Hu et al. reported that 24,447 ovarian lncRNAs associated with prolificacy of Large White sows were identified during the follicular and luteal phases of the estrous cycle (Hu et al., 2020). In the follicular development, La et al. (2019) found that 473 lncRNAs were differentially expressed by comparing polytomous and monotocous Small Tail Han sheep (Ovis aries). Peng et al. (2019) identified 550 lncRNAs that differ in follicles between two different chicken breeds. Yak, an important animal in the economics in the Qinghai-Tibet Plateau, is one of the bovine animals with strong adaptability to the low oxygen environment and is known as the boat of the plateau (Yao et al., 2018). However, the lncRNAs expression pattern in yak healthy and atretic follicle has not been identified.

In this study, RNA-seq was used to identify the expression profile of lncRNAs, miRNAs, and mRNAs in healthy and atretic follicles. A regulatory network of lncRNA/miRNA/mRNA was constructed.

## Methods

### Animals

A total of 10 female yaks weighing 250–300 kg and of 6–7 years of age were selected from the yak farm belonging to the National Research Centre on Yak, situated 2750 m above sea level in Linzhi, Tibet. The animals were slaughtered and healthy yak ovaries were obtained. The animals were healthy and free from any anatomical re-productive disorders and completed 2 years of the postpartum period. The yak ovaries were harvested and stored in physiological saline at 38°C before experimental analyses. Healthy and atretic follicles were separated according to our previous method (Yao et al., 2018).

### Institutional review board statement

All experiments were conducted by the guidelines of the regional Animal Ethics Committee and were approved by the Institutional Animal Care and Use Committee of Xi Zang Agricultural and Animal Husbandry College. The institutional certification number is 12540000MB0P013721.

### RNA isolation, library preparation, and sequencing

Total RNA was extracted from the three healthy and three atretic follicle using Trizol reagent (Invitrogen, Life Technologies, Carlsbad, CA, United States) by the manufacturers’ instructions. RNA purity was measured using a NanoDrop 2000 (NanoDrop Technologies, Wilmington, DE, United States), and RNA integrity and concentration were determined with an Agilent 2100 Bioanalyzer (Agilent Technologies, Santa Clara, CA, United States). Ribosomal RNA (rRNA) was removed using a Ribo-Zero Magnetic Gold Kit (Epicentre, Madison, WI, United States). Subsequently, the two cDNA libraries were prepared with an NEB Next Ultra Directional RNA Library Prep Kit for Illumina (NEB, Ipswich, MA, United States) by the manufacturer’s instructions. The libraries were then sequenced on a HiSeqXten platform with 150 bp paired-end reads (Illumina, San Diego, CA, United States).

### Data analysis

Data analysis was performed according to Venø et al. (2015) with minor modifications. Briefly, paired-end reads were harvested from the Illumina HiSeq 4000 sequencer; quality control was performed by Q30 after 3’ adaptor-trimming and the removal of low-quality reads by Fastp software (v1.9.3). The high-quality trimmed reads were used to analyze miRNAs, mRNAs, and lncRNAs, respectively.

For lncRNA data analysis, the high-quality reads were aligned to the cattle reference genome (Sscrofa10.2) using Hisat2 software (v2.0.4). Then, guided by the Ensembl gtf gene annotation file, Cuffdiff software was used to obtain the FPKM as expression profiles of lncRNA. Fold change and *p*-value were calculated based on FPKM and differentially expressed lncRNAs were identified. LncRNA potential target genes were predicted by their locations to nearby genes.

LncRNA target genes predicted: According to the nucleotide sequence characteristics of the novel lncRNA, we use Starbase to predict and summarize the target genes, and use the Phatmap R package to draw the heat map.

Heat map: We extracted the expression of TOP4 gene (lncRNA, miRNA and mRNA) in different follicles, mapped it with Phatmap R package, and homogenized it with Z-score.

### GO and pathway enrichment analysis

Gene Ontology analysis for host genes of different expression lincRNAs by Gene Ontology terms1 was conducted using the Blast2GO program2 (Conesa et al., 2005) with an E-value cut-off at 10−5. Pathway functional annotation for host genes of different expression lncRNAs was performed through sequence comparisons against the Kyoto Encyclopedia of Genes and Genomes (KEGG) database (Kanehisa Laboratories, Kyoto, Japan)3 using the BLASTX algorithm (E-value threshold: 10−5). GO terms and pathways enrichment analysis was performed with the hypergeometric test, and a Benjamini-Hochberg method corrected *p* -value ≤ 0.05 was considered to significantly enriched GO terms and pathways.

### qRT-PCR

Total RNA was extracted from the healthy follicular and used in the RNA-seq and reversed to complementary DNA (cDNA) with a Primescript RT Master Kit (Takara, Dalian, China) with random primers by the manufacturer’s instructions. Then, qRT-PCR was performed on a 7500 FAST Real‐Time PCR System (Applied Biosystems) according to the SYBR Premix Ex Taq TM instructions. The cycling conditions for qRT- PCR were as follows: 50 °C for 2 min; 95 °C for 2 min; and 40 cycles of 95 °C for 15 s, 60 °C for 1 min, and 95 °C for 15 s. Each reaction was performed in a 20 μL reaction mixture containing 10 μL of ChamQ SYBR qPCR Master Mix (Vazyme Q311-02, Nanjing, China), 0.5 μL of gene‐specific primers, 2 μL of template cDNA, and 7 μL of sterile water. All reactions were performed in triplicate for each sample. The expression level of lncRNAs was normalized to the reference gene glyceraldehyde-3- phosphate dehydrogenase (GAPDH), and the relative expression level of the lncRNAs was calculated via the 2^-△△CT^ method. miRNA reverse primer from the qRT-PCR kit (Vazyme MQ101-01, Nanjing, China). The primer sequence was listed in Table 1.

**Table 1:** Primers in this study.

**Name**	**Primer sequences (5'-3')**	**Application**
MSTRG.19263.1 WT	F: CGAGCTCGACCAAACTGACAACATGTGTAGAG	Vector construct for miR-26b binding sites
	R: CCTCGAGTGCCCTGTCTTTCCCTCCATTGTTT'	
MSTRG.19263.1 MT	F: AACTGACAACATGTGTAGAGGAAAATGACAAGGAT	Mutation Vector construct
	R: TGGTCTGTATGTTTTCAAGTATTTCATGGCCCAGG	
MSTRG.19263.1 WT	F: CGAGCTCCATATATATGCATTAGTATATGACA	Vector construct for miR-378 binding sites
	R: CCTCGAGCTTTGTTTTCTTGTTACCAGTAAGA	Mutation Vector construct
MSTRG.19263.1 MT	F: GAATGTCATTTTCTTAAGACAGATTAAACCAATAT	
	R: TGATATTTCCTCCTGGACTTAACCAGTTCAATT	
SEMA6D 3’UTR WT	F: CGAGCTCTTCTTTTGTTTGAAGCTAAAGAGAT	Vector construct
	R: CCTCGAGATCTCTTTAGCTTCAAACAAAAGAA	
SEMA6D 3’UTR MT	F: TAGACTGCCATTTTGTGTGGTCTTCCCATTAAATG	Mutation Vector construct
	R: AGTTGAACCCATTTTCAAGTATTTGCTCACAGACA	
ARL6 3’UTR WT	F: CGAGCTCATGAAAGAAACAGAAGGCAAAAGGT'	Vector construc
	R: CCTCGAGACCTTGTATCCTCATCAAAACCATT	
ARL6 3’UTR MT	F: TTTGGCAAATTGAAAATTACCCAGACTATTCCAGT	Mutation Vector construct
	R: ATTACTGGGACTTCTGGACTAAGAGAAACTGGATT	
MSTRG.26418.1	F: TAGAGGGGTGGGACTTGCCTGGTGG	qRT-PCR
	R: ACAGAGCTAAGATCCTATATATACA	
MSTRG.21442.4	F: TAGGCTCTGGAGAAGGCAATGGCAC	qRT-PCR
	R: CCCATTCCTATCTAACTCCCTTTTC	
MSTRG.20814.1	F: CAAGGCAAGAATACTGAAGTGGTTT	qRT-PCR
	R: TTGCCAACAAAGGTCCGTATAGTCA	
MSTRG.19758.2	F: ATAAGATTATTTGCAACTATTCCTC'	qRT-PCR
	R: TGTGTATTTAACACCCCATCTTCCT	
Bta-let-7d	F: TGAGGTAGTAGGTTGTATGGTT	qRT-PCR
Bta-miR-493	F: ACTGGACTTGGAGTCAGAAGGC	qRT-PCR
Bta-miR-26b	F: TTCAAGTAATCCAGGATAGGCT	qRT-PCR
Bta-miR-182	F: TTTGGCAATGGTAGAACTCACACT	qRT-PCR
SIRT4	F: GACGATAGCAAAGCAAATTCAGATG	qRT-PCR
	R: ATGAAGCCCAAGATGTTTTCATGCC	
DAP	F: TATGACTTCAAAGCCACCGCAGACG	qRT-PCR
	R: GATGAAGCCGTCTTTCCCATTGAGC	
DCLK1	F: CCTTGGAAGAGAGTTACAAAATGGA	qRT-PCR
	R: GGCTTAGAAGCACACAAATAAAACT	
PALM	F: TGATGAATGCAAACGAAGATTTAAT	qRT-PCR
	R: GCAGACAGTTGAAACAATCAGTGAA	

### Target MiRNAs and genes prediction, and network analysis

Yak miRNA sequences were obtained from the miRBase database and the binding sites of miRNA in lncRNAs and genes were predicted using Miranda with a strict model. The 3’- untranslated region (UTR) sequences were downloaded from the UCSC Genome Browser4. The co-expression network of lincRNA-miRNA-mRNA was constructed using Cytoscape software. In brief, we import the differential genes into the official website of String (2021), and then import the correlation table into Cytoscape software to draw the network. The miRNA sequence was listed in Table 2.

**Table 2:** Small fragments of RNA synthesized in the present study.

**Name**	**Sequences (5'-3')**
Bta-miR-378 mimics	ACUGGAACUUGGAGUCAGAAGGC
Bta-miR-26b mimics	UUCAAGUAUUCAGGAUAGGAA
mimics NC	UUGUACUACACAAAAGUACUG

### Transfection and dual-luciferase assay

The bta-miRNA mimics were purchased from GenePharma (Shanghai, China). For the dual-luciferase assay, sequences of *ARHGEF28: MSTRG.19263.1*, *SEMA6D*, and *ARL6* containing a bta-miR-26b, bta-miR-378 binding site were synthesized by TSINGKE Company (Nanjing, China) and inserted into the *Nhe* I/*Sal* I site in the pmirGLO Dual-Luciferase report vector. HEK293T cells were grown to 75% to 80% confluence in 12-well plates and then co-transfected with a vector and miRNA using Lipofectamine 3000 reagent (Invitrogen) according to the manufacturer’s instructions. The cells were harvested after 24 hours, and luciferase activity was evaluated using a dual-luciferase assay system (Promega).

### Statistical analysis

Statistical analyses of dual-luciferase assay and Pearson correlation coefficient were performed using SPSS 20.0 (SPSS Inc., Chicago, IL, United States). Results are expressed as the mean ± SEM, and statistically significant differences between the two means were analyzed using Student’s *t*-test. A value of *p* < 0.05 was considered statistically significant.

## Results

### Identification of healthy follicles and atretic follicles in yaks

**Figure 1:**
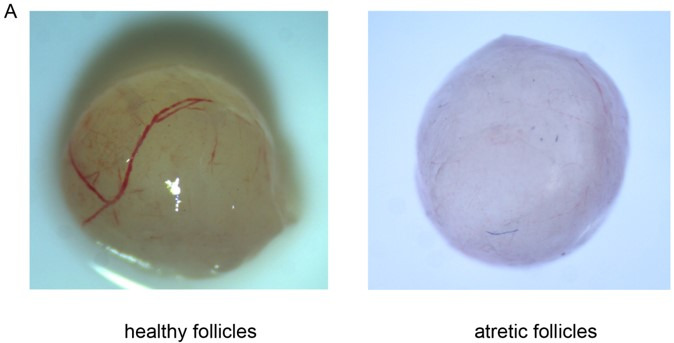
Morphological criteria for follicle classification. Left panel is the health follicle; the right panel is the atretic follicle.

To understand the regulatory factors of yak follicular development. We isolated healthy and atretic follicles. The appearance of healthy follicles is clear and pink. Cumulus oocyte complex can be seen on the follicle wall. The granular cell layer is complete, compact, and uniform. The appearance of atretic follicles is turbid and light gray, the cumulus-oocyte complex falls off into the follicular cavity, the granular cell layer falls off seriously, and there are a large number of turbid fragments in the follicular cavity (Figure 1).

### Differentially expressed RNAs distinguish between yak healthy and atretic follicle

**Figure 2:**
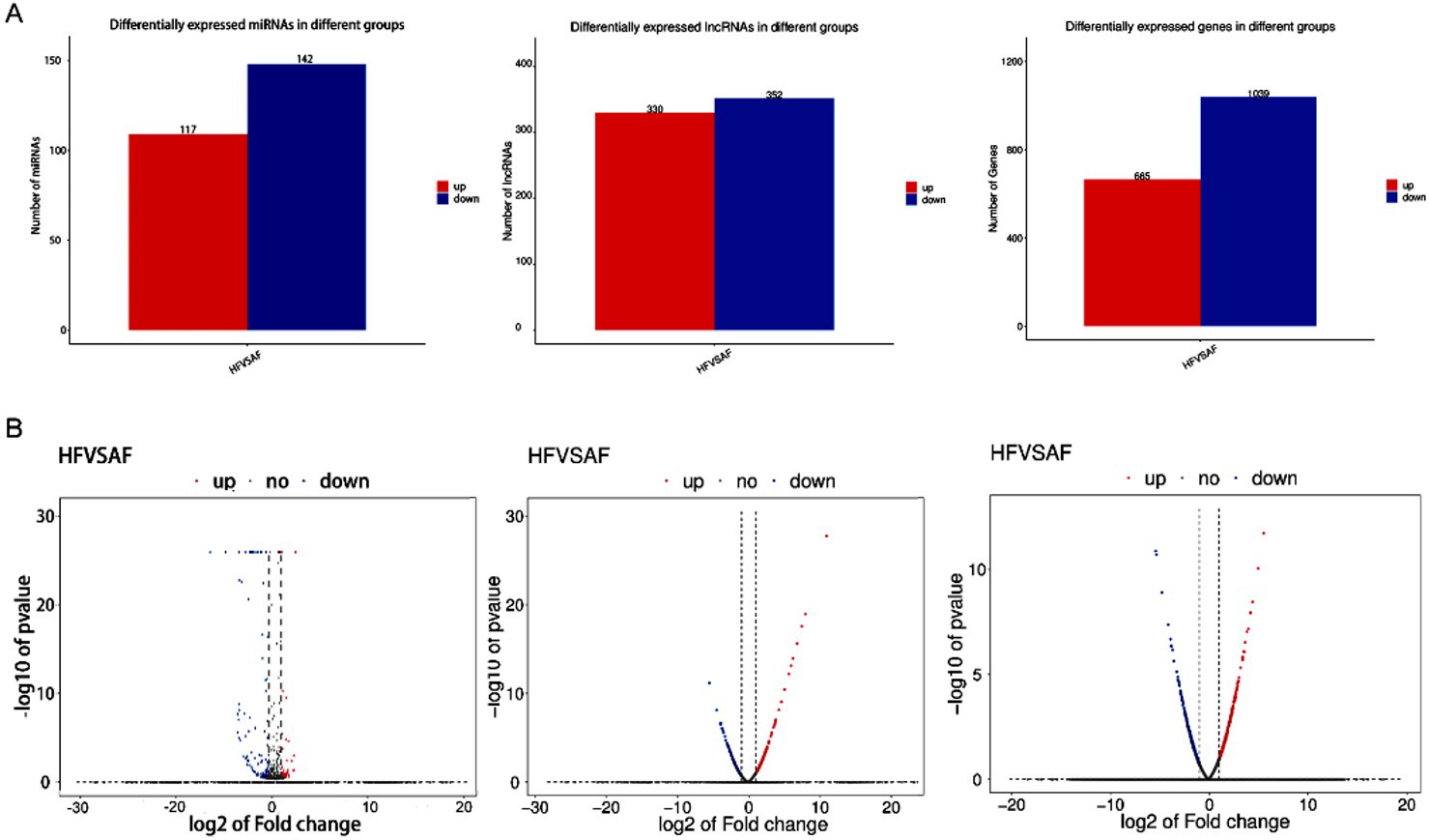
Barplots and volcano plots presenting differentially expressed lncRNAs, miRNAs, and mRNAs. (A) Barplots for all differentially expressed miRNAs, lncRNAs, and mRNAs between healthy and atretic follicles; (B) Volcano plots showing miRNAs, lncRNAs, and mRNAs with fold change ≥ 2 (*p* < 0.001). Blue, downregulated; blue, upregulated; gray, not differential expressed.

Using RNA-seq, we have detected numerous transcripts in yak follicles of healthy and atretic. Of these, A total of 682 novel lncRNAs, 1704 mRNAs, and 259 bta-miRNAs were identified as being differentially expressed between healthy and atretic follicles with consider-ing |log 2fold change|>1 and an adjusted FDR of *p* < 0.05 (Supplementary Tables 1, 2, and 3). Among them, 352 lncRNAs were upregulated and 330 were downregulated, 665 mRNAs were upregulated and 1039 downregulated, 142 bta-miRNAs were upregulated, and 117 downregulated in healthy versus atretic follicles (Figure 2A-2B).

### Comparison of lncRNA and mRNA characteristics

**Figure 3:**
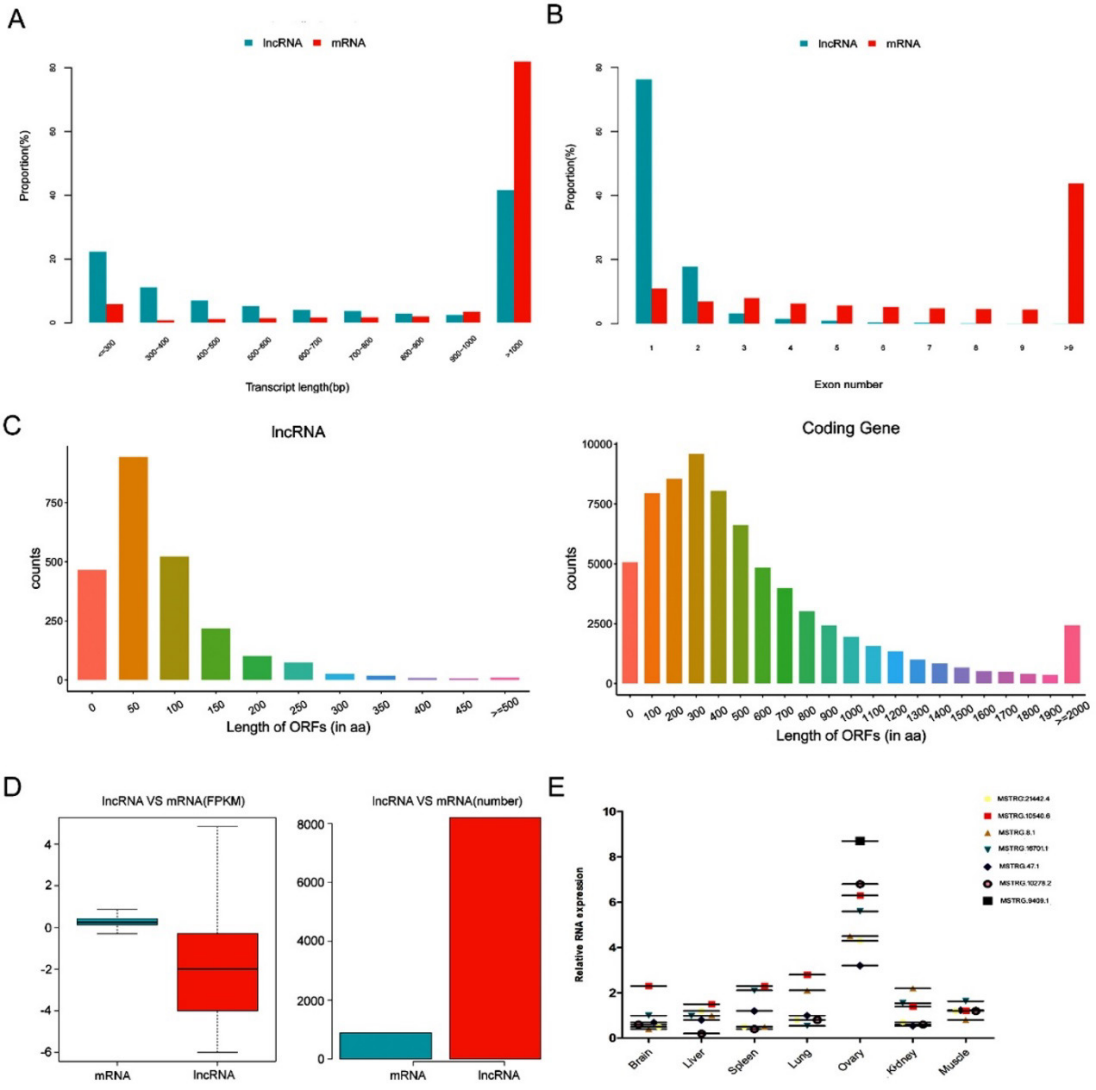
Characterization of long non-coding RNAs (lncRNAs) and messenger RNAs (mRNAs) from antral follicle granulosa cells. (A-C), the transcript length, exon numbers and length of ORFs of novel lncRNAs and mRNAs. (D) Bar plot and density distribution diagram showing the expression features of lncRNAs and mRNAs in granulosa cells from healthy and atretic follicles, respectively. FPKM, fragments per kilobase million. (E) Tissue expression profile of different lncRNAs expression in different tissues (brain, liver, spleen, lung, ovary, kidney).

We described the characteristics of 682 novel lncRNAs and 1704 mRNAs. Our results indicated that lncRNAs transcripts were shorter than mRNAs (Figure 3A); their genes tended to contain fewer exons (Figure 3B). The length of lncRNA ORFs was also shorter than mRNAs (Figure 3C). Furthermore, the expression levels and the numbers were lower than mRNAs (Figure 3D). Our results were observed to be consistent with previous studies (Ling et al., 2019). In addition, the characteristic of lncRNAs is that they exhibit obvious tissue specificity. So, we next examined the expression of randomly selected lncRNAs in 7 tissues. The results showed that the lncRNA presented obvious tissue specificity expression in the ovary (Figure 3E).

### GO and KEGG enrichment analyses of differentially expressed lncRNAs

**Figure 4:**
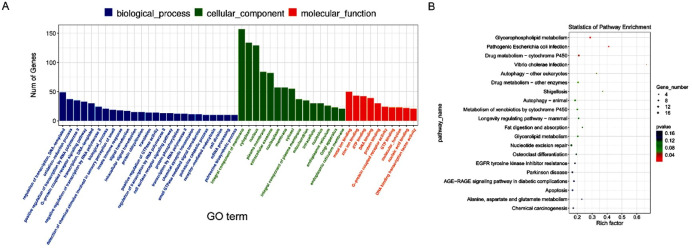
GO and KEGG enrichment analysis of host genes of different expression lncRNAs. (A) GO categories (biological process) of differential lncRNA target genes in healthy and atretic follicles. (B) The most significantly enriched pathways of host genes of different expression lncRNAs. The size and color of each bubble represent the number of genes in each pathway and *P* value respectively.

In order to clarify the function of novel differentially expressing lncRNA between yak healthy and atretic follicles. We performed GO term and KEGG pathway enrichment analyses on the target genes of lncRNA. The results revealed that 50 GO terms were significantly enriched in the biological process, cellular component, and molecular function, respectively (Figure 4A, Supplementary Tables 4-5). Pathway enrichment analysis showed that the host genes were significantly enriched in 12 pathways, including the development of follicular granulosa cells-related signaling pathways, such as autophagy and apoptosis pathways (Figure 4B). In addition, the correlation between lncRNAs and target mRNAs also were analyzed, and the results showed that Nine mRNAs were positively correlated with 10 lncRNAs, and 11 mRNAs were negatively correlated with 12 lncRNAs (Figure S1). It is indicated that different lncRNA regulates yak follicular development may through different mechanisms.

### LncRNAs, miRNAs and mRNAs differentially expressed between yak healthy and atretic follicles

**Figure 5:**
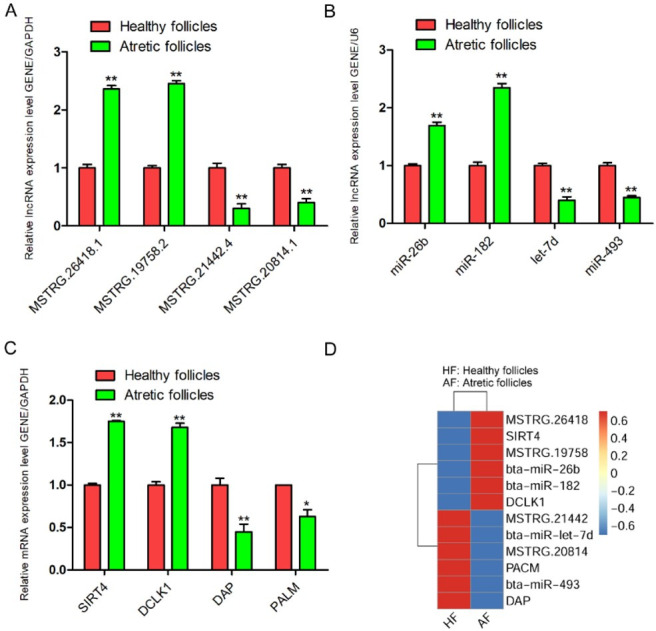
The levels of lncRNAs (A), miRNAs (B) and mRNAs (C) expression in healthy and atretic follicles using qRT-PCR. (D) Heat-map results for lncRNA, miRNA and mRNA expression. *GAPDH* as a reference gene. Experiments were performed in triplicate, * *p* < 0.05, ** *p* < 0.01.

To verify the RNA-seq results, four lncRNAs *(MSTRG.26418.1*, *MSTRG.21442.4*, *MSTRG.19758.2* and MSTRG.20814.1) and miRNAs (bta-miR-182, bta-miR-493, bta-miR-26b and bta-let-7d) were selected for qRT-PCR. The selected lncRNAs and miRNAs were consistent with the results of RNA-sequencing (Figure 5A-5B, Figure S2A). And, four mRNAs also were randomly selected to verify the accuracy of the mRNA-related data (Figure 5C, Figure S2B). The results showed that *SIRT4* and *DCLK1* were highly expressed in atretic follicles. The *DAP* and *PALM* were highly expressed in healthy follicles. These results were also consistent with RNA-seq results. And, the results of RNA-seq were also shown in Figure 5.

### The ceRNA network is a biological network performing functions

**Figure 6:**
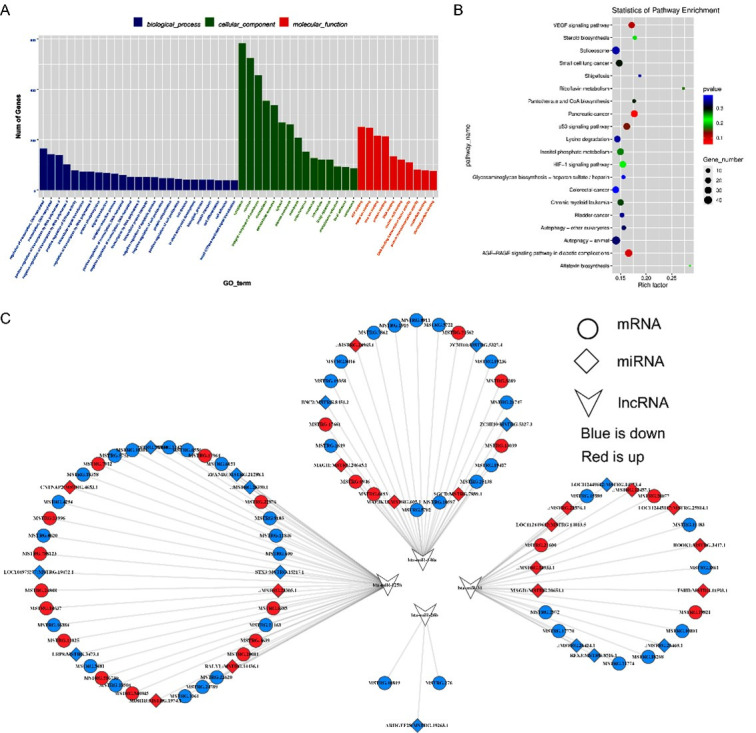
GO and KEGG pathway enrichment analysis of different expressed mRNAs associated with the ceRNA network. (A) GO analysis of differentially expressed genes. (B) The most significantly enriched pathways of differential expression genes. The size and color of the bubble represent the number of genes enriched in the pathway and enrichment significance, respectively. (C) The ceRNA network derived from different expression genes indicates lncRNAs.

In order to lay a foundation for exploring the regulatory mechanism of lncRNA in yak follicles. We performed ceRNA interaction analysis on the differentially expressed lncRNAs (Supplementary Table 6). Furthermore, GO and KEGG analyses were performed on the related genes in the ceRNA network. The GO analysis revealed that a number of genes were significantly associated with cell adhesion, cell proliferation, and cell surface processes (*p* < 0.01) (Figure 6A, supplementary Table 7). In line with these results, KEGG pathway analysis made clear that the targeted transcripts were primarily linked to apoptosis-related pathways including the MAPK signaling pathway and insulin-resistant pathway (Figure 6B, supplementary Table 8). Moreover, we showed that bta-miR-125b (Yao et al., 2018), bta-miR-31 (Zhang et al., 2019b), bta-miR-378 (Sun et al., 2018), and bta-miR-146a (Chen et al., 2015) ceRNA network interacting with lncRNAs and mRNAs. As shown in Figure 6C, 390 lncRNA were associated with miR-125b, 140 lncRNAs were associated with miR-146a, and 4 lncRNAs were associated with miR-26b, and 140 lncRNAs were associated with miR-31. The construction of the ceRNA network of these lncRNAs will point out the direction for the next step to explore the regulation of lncRNA/miRNA/mRNA in yak follicle development.

### Functional Analysis of lncRNA as a ceRNA

**Figure 7:**
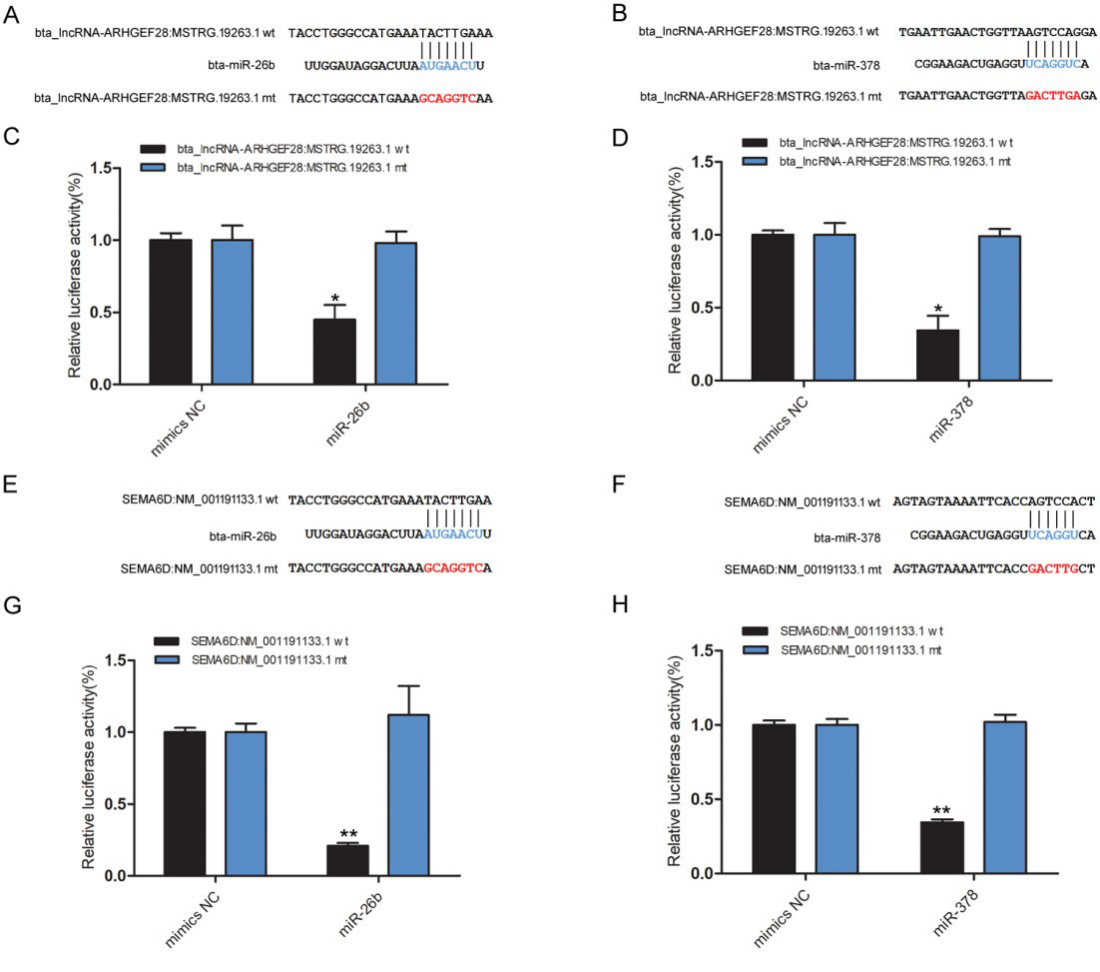
The binding sites predicted and dual-luciferase activity assays. (A–B) The predicted binding site and mutated site of bta-miR-26b and bta-miR-378 in *MSTRG.19263.1*. (C–D) Luciferase assay using reporter constructs with wild-type (WT) or mutant (Mut) of *lincRNA ARHGEF28: MSTRG*. HEK293T cells in 24-well plates were transfected with the WT or Mut luciferase reporters of *MSTRG.19263.1*, along with bta-miR-26b and bta-miR-378 mimics or control. (E–F) The predicted binding site and mutated site of bta-miR-26b and bta-miR-378 in *SEMA6D* and *ARL6*, respectively. (G–H) Luciferase assay using reporter constructs with wild-type (WT) or mutant (Mut) of *SEMA6D* and *ARL6* 3’UTR. Data are shown as the mean ± SEM (n = 3). Experiments were performed in triplicate, * *p* < 0.05, ** *p* < 0.01.

Given that *ARHGEF28: MSTRG.19263.1* acts as bta-miR-26b and bta-miR-378 sponges, we next investigated the binding capability of the *MSTRG.19263.1* to bta-miR-26b and bta-miR-378 using the dual-luciferase assay. Figures 7A-7B shows the predicted binding site and mutated site of bta-miR-26b and bta-miR-378 in *MSTRG.19263.1*. The lincRNAs dual-luciferase reporter vectors were constructed and co-transfected into HEK293T cells with the bta-miR-26b and bta-miR-378 mimics or control. The results showed that bta-miR-26b and bta-miR-378 significantly reduced the luciferase activity of wild-type luciferase reporters of *MSTRG.19263.1* compared to control (Figures 7C-7D), whereas, bta-miR-26b and bta-miR-378 had no effect on the mutated luciferase reporters. These results suggest that *MSTRG.19263.1* can bind to and function as sponges for bta-miR-26b and bta-miR-378.

In addition, the mRNA also plays important role in the ceRNA mechanism, we also randomly selected bta-miR-26b and bta-miR-378 predicted target genes (*SEMA6D*: NM_001191133.1 and *ARL6*: NM_001075782.1) to verify the binding capacity. We have predicted the miR-26b and miR-378 binding sites on *SEMA6D* and *ARL6*, respectively (Figures 7E-7F). The target genes dual-luciferase reporter vectors were constructed and co-transfected into HEK293T cells with the bta-miR-26b and bta-miR-378 mimics or control. The results showed that bta-miR-26b significantly reduced the luciferase activity of wild type luciferase reporters of *SEMA6D* compared to control, whereas, had no effect on the mutated luciferase reporters. The bta-miR-378 significantly reduced the luciferase activity of wild type luciferase reporters of *ARL6* compared to control (Figures 7G-7H), whereas, had no effect on the mutated luciferase reporters. These results indicate that our ceRNA network is successfully constructed.

## Discussion

Previous studies have shown that lncRNAs act as key post-transcriptional regulators in animals (Li et al., 2015). In this study, we identified lncRNAs from healthy follicles and atretic follicles of yak for the first time, which may play an important role in the development of yak follicles. Our results show that lncRNAs in yak follicles are produced at different genomic locations (intergene exon introns and antisense), for example non-coding region of the gene coding region exon intron justice chain or antisense chain, which is consistent with previous findings (Ponting et al., 2009). In addition, combined with mRNA and lncRNA expression profiles, we conducted multi-level analysis to study how the differences in lncRNA, miRNA and mRNA expression profiles lead to follicular development. A total of 882 lncRNA transcripts were observed differentially expressed in healthy follicles and atretic follicles. The RNA-seq data were further confirmed by qRT-PCR. The potential target genes of lncRNA were detected by KEGG analysis, development of follicular granulosa cells-related signaling pathways, such as autophagy and apoptosis pathways.

At present, reported on the function of lncRNA in follicular development mainly focus on the physiological or pathological aspects of follicular growth. For example, *lncRNA-BANCR* inhibits proliferation and induces apoptosis in KGN cells (Yang et al., 2019), whereas *lncRNA-HCP5* promotes cell proliferation and inhibits apoptosis by interacting with miR-27a-3p/*IGF-1* axis(Chen et al., 2020). There are limited reports on the role of lncRNAs in ovaries and follicles development. In particular, transcriptome sequencing and combined analysis of mRNA, miRNA and lncRNA between healthy and atretic follicles of yaks have not been reported. In order to more fully understand the genes involved in follicular atretic, we further analyzed the changes of differential mRNA transcriptome profiles in healthy and atretic follicles of yaks. RNA-seq data showed that there were 1704 differentially expressed genes in healthy follicles and atretic follicles, among which 1039 differentially expressed genes were higher and 665 differentially expressed genes were lower. KEGG pathway analysis revealed that genes up-regulated in atretic follicles were highly enriched during inflammation and apoptosis. At the same time, we noted that the expression of *FSHR* was decreased during follicular atretic in yaks. The important role of follicle-stimulating hormone (*FSH*) in the maturation of mammalian oocytes in vitro has long been demonstrated (Zhao et al., 2020). In animals, *FSH* mainly stimulates follicular growth (Glick et al., 2013). However, *FSH* is usually mediated by a specific receptor *FSHR* distributed in granulosa cells (Matsui et al., 2004). During follicular development, the expression of *FSHR* remained unchanged in healthy cavitary follicles, but decreased during follicular atretic (Du et al., 2016). Moreover, we also identified that the expression of *MSTRG.11593.1* was decreased during follicular atretic in yaks. It is indicated that lncRNA may be involved in granulosa cell apoptosis, leading to follicular atretic. And, the exact effect of lncRNA on granulosa cell apoptosis and its role in follicular development need to be further studied.

MicroRNAs (miRNAs) are a class of small non-coding RNAs that can regulate gene expression after transcription by binding to targets (Kim et al., 2006). Studies have shown that lncRNAs bind to miRNAs as competitive endogenous RNAs (ceRNAs) (Luo et al., 2019; Dai et al., 2019; Luan and Wang, 2018). For example, *lncRNA-CDC6* can act as a sponge for miR-215 promotes breast cancer progression (Kong et al., 2019). *Claudin-4* acts as a sponge of miR-596 and miR-3620-3p and reinforce proliferation, invasion, and EMT in AGS, HGC-27, and SGC-7901 cells (Song et al., 2017). In this study, we also performed transcriptome analysis of miRNA in healthy follicles and atretic follicles, and identified 269 differentially expressed lncRNAs. As miRNAs are highly conserved among species, we conducted co-expression network analysis for reported miRNAs involved in follicular development. The results showed that *MSTRG.11593.1* was bound to bta-miR-31. Interestingly, miR-31 has been reported to regulate apoptosis and estrogen secretion of follicular granulosa cells by affecting the expression of *FSHR*. *FSHR* and *MSTRG.11593.1* were both down-regulated in atretic follicles of yaks. We speculate that *FSHR* may exist in follicular atretic: the ceRNA mechanism of MSTRG.11593.1/miR-31/FSHR regulates follicular atretic in yaks. In addition, bta-miR-26b and bta-miR-378 target *MSTRG.19263.1*. MiR-26b and miR-378 have been reported to be important miRNAs in the development and maturing of porcine follicles (Sun et al., 2018; Liu et al., 2014). Expression profiling analysis showed that *MSTRG.19263.1* in healthy follicles was higher than that in atretic follicles, and the expression pattern of miR-26b and miR-378 in follicles was opposite to that of target lncRNAs. In addition, dual-luciferase assay results confirmed that miR-26b and miR-378 could bind to *MSTRG.19263.1*. These results suggest that *ARHGEF28: MSTRG.19263.1* may regulate follicular development through interactions with miR-26b and miR-378. Therefore, the results of co-expression network analysis provide a possible mechanism for regulating follicular maturation and atretic, although the specific regulatory procedures still need to be identified.

## Conclusion

We identified differentially expressed lncRNAs, miRNAs, and mRNAs in healthy and atretic follicles of yak. Among them, 330 lncRNAs were highly expressed in healthy follicles, and 352 lncRNAs were highly expressed in atretic follicles. KEGG analysis found that differentially expressed lncRNAs were associated with hormone secretion, granulosa cell apoptosis, and follicle development. In addition, we also constructed a ceRNA interaction network of differentially expressed lncRNAs. These results lay the foundation for further studies on the role of lncRNAs in yak follicle development.

## Data availability statement

Data are available from the corresponding author upon request. Transcriptome and miRNA sequence data have been deposited in NCBI SRA (accession codes SUB9750825 and SUB9765662 respectively).

## Supplementary Material

Supplementary material accompanies this paper.

Table 1 Supplementary. Different expression lncRNAs

Table 2 Different expression mRNAs

Table 3 Different expression miRNAs

Table 4 GO analysis for host genes of lncRNAs

Table 5 KEGG analysis for host genes of lncRNAs

Table 6 ceRNA network constructed of different expression lncRNAs

Table 7 GO analysis for miRNAs binding genes

Table 8 KEGG analysis for miRNAs binding genes

Figure S1 The correlation between lncRNAs and target genes. Bule is negative correlation, red is positive correlation

Figure S2 Genes expression in Healthy and Atretic follicles. (A-B) LncRNA and mRNA expression were determined in Healthy and Atretic follicles. *β-catin* as a refernce gene. Experiments were performed in triplicate, * *p* < 0.05, ** *p* < 0.01.

This material is available as part of the online article from 10.1590/1984-3143-AR2021-0131
