# Supplementary material for: Profiling and Functional Analysis of long non-coding RNAs in yak healthy and atretic follicles
Source: Anim Reprod. 2022 Oct 24;19(3):e20210131. doi: 10.1590/1984-3143-AR2021-0131 (PMC9613354; doi:10.1590/1984-3143-AR2021-0131)
Supplement: Figure S1 [file 1984-3143-ar-19-3-e20210131-suppl02.pdf]

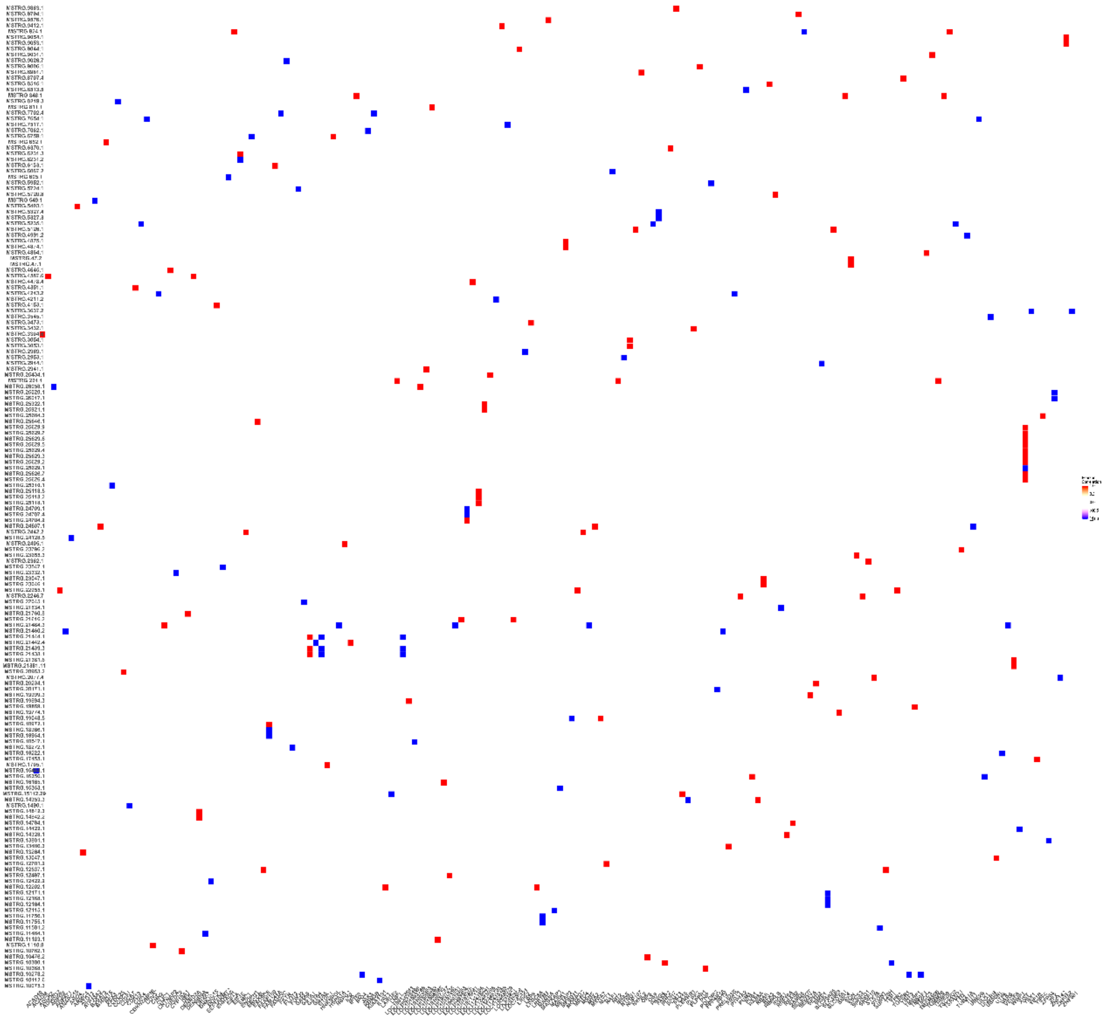

Figure S1 The correlation between lncRNAs and target genes. Bule is negative correlation, red is positive correlation.

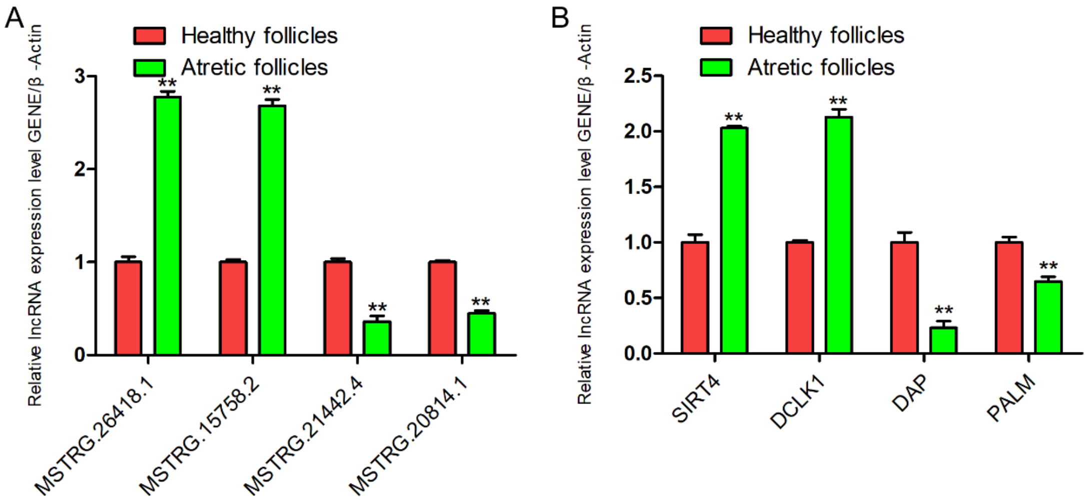

Figure S2 Genes expression in Healthy and Atretic follicles. (A-B) LncRNA and mRNA expression were determined in Healthy and Atretic follicles. *β-catin* as a refernce gene. Experiments were performed in triplicate, * $p < 0.05$, ** $p < 0.01$.
